# Impact of long‐lasting spontaneous physical activity on bone morphogenetic protein 4 in the heart and tibia in murine model of heart failure

**DOI:** 10.14814/phy2.14412

**Published:** 2020-04-21

**Authors:** Joanna Majerczak, Joanna Filipowska, Grzegorz Tylko, Magdalena Guzik, Janusz Karasinski, Ewa Piechowicz, Elżbieta Pyza, Stefan Chlopicki, Jerzy A. Zoladz

**Affiliations:** ^1^ Department of Neurobiology Faculty of Health Sciences Poznan University of Physical Education Poznan Poland; ^2^ Department of Translational Research and Cellular Therapeutics City of Hope Duarte CA USA; ^3^ Department of Cell Biology and Imaging Institute of Zoology and Biomedical Research of the Jagiellonian University Krakow Poland; ^4^ Department of Muscle Physiology Faculty of Rehabilitation University School of Physical Education Krakow Poland; ^5^ Jagiellonian Centre for Experimental Therapeutics Jagiellonian University Medical College Krakow Poland; ^6^ Department of Pharmacology Jagiellonian University Medical College Krakow Poland

**Keywords:** BMP4, heart failure, physical activity

## Abstract

Bone morphogenetic protein 4 (BMP4) plays an important role in bone remodeling and in heart failure pathogenesis. The aim of this study was to evaluate the effect of spontaneous physical activity on the expression of BMP4 in the heart and tibia of the transgenic (Tgαq*44) mice, representing a model of chronic heart failure. Tgαq*44 and wild‐type FVB mice (WT) were randomly assigned either to sedentary or to trained groups undergoing 8 weeks of spontaneous wheel running. The BMP4 protein expression in heart and tibiae was evaluated using Western immunoblotting and the phosphorus and calcium in the tibiae was assessed using the X‐ray microanalysis. BMP4 content in the hearts of the Tgαq*44‐sedentary mice was by ~490% higher than in the WT‐sedentary mice, whereas in tibiae the BMP4 content of the Tgαq*44‐sedentary mice was similar to that in the WT‐sedentary animals. Tgαq*44 mice revealed by ~28% poorer spontaneous physical activity than the WT mice. No effect of performed physical activity on the BMP4 content in the hearts of either in the Tgαq*44 or WT mice was observed. However, 8‐week spontaneous wheel running resulted in a decrease in the BMP4 expression in tibiae (by ~43%) in the group of Tgαq*44 mice only, with no changes in their bone phosphorus and calcium contents. We have concluded that prolonged period of spontaneous physical exercise does not increase the risk of the progression of the BMP4‐mediated pathological cardiac hypertrophy and does not affect bone mineral status in the chronic heart failure mice.

## Graphical abstract

This study shows that long‐lasting physical exercise does not increase the risk of the progression of the BMP4–mediated pathological cardiac hypertrophy. Moreover, assuming the pro‐inflammatory function of BMP4, training‐induced decrease in the BMP4 content in bone might have a slowing down impact on the progression of heart failure.

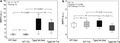

## 1 INTRODUCTION

Heart failure (HF) is a clinical syndrome characterized by a progressive deterioration of the cardiac function, leading to a decrease in capacity of the heart to meet the body requirements for blood flow (Grassi et al., 2017; Poole, Richardson, Haykowsky, Hirai, & Musch, 2018). Heart failure patients usually struggle with low exercise tolerance, which is related not only to a diminished heart function, but also to the skeletal muscle weakness (Okita, Kinugawa, & Tsutsui, 2013; Szentesi et al., 2005) and early muscle fatigue (Grassi, Rossiter, & Zoladz, 2015). Moreover, this disease is associated with a higher risk of bone loss and thus greater predisposition to bone fractures (Jankowska et al., 2009).

It is well established that a regular physical activity exerts beneficial effects in muscles (Baldwin & Haddad, 2019) and bones (Kemmler & von Stangel, 2019). One of the most prominent effects of the endurance training on skeletal muscles is an increase in the mitochondrial enzyme activity (Holloszy, 1967), mitochondrial content (Hoppeler et al., 1985), and muscle mitochondria efficiency (Zoladz, Koziel, Woyda‐Ploszczyca, Celichowski, & Jarmuszkiewicz, 2016), leading to an increase in the metabolic stability during exercise and enhancement of exercise tolerance (Dudley, Tullson, & Terjung, 1987; Korzeniewski & Zoladz, 2004; Zoladz, Grassi, & Szkutnik, 2019; Zoladz, Korzeniewski, & Grassi, 2006). At the heart muscle level, the endurance training has been shown to increase the activity/content of mitochondrial proteins (Momken, Lechêne, Ventura‐Clapier, & Veksler, 2004) and mitochondrial volume and density (Vettor et al., 2014).

Regarding the skeleton strength, physical exercise promotes it mainly by prevention of bone loss (Robling & Turner, 2009) and increase in bone mineral density (Boudenot et al., 2015; Trabelsi et al., 2016). Mechanical stimulation triggering bone cells anabolic response via mechanotransduction is indispensable not only for proper skeleton development during growth but also for bone remodeling during exercise (Robling & Turner, 2009). The Wolf's law and Frost's mechanostat theory (Frost, 1994) were the first ones to explain skeleton adaptation to mechanical loading and its significance in bone homeostasis. The exact mechanisms of mechanotransduction are still subject to debate, however, transforming growth factor β superfamily/ bone morphogenetic proteins signaling pathway (TGF‐β/BMP) (Siamwala, Rajendran, & Chatterjee, 2015) and Wingless‐Int1/β catenin signaling pathway (Wnt/β catenin) (Robling & Turner, 2009) are the main signaling pathways most likely implicated in exercise‐induced bone strengthening (Chen et al., 2016; Hu, Yang, Wu, & Liu, 2019). Besides enhanced mechanical stimuli (muscular tension and axial loading) also systemic changes, that is, an increase in free testosterone levels, 17‐β‐estradiol, human growth hormone (hGH), insulin‐like growth factor 1 (IGF‐1), parathyroid hormone (PTH), calcitriol contribute to skeleton strengthening under exercise (Kemmler & von Stangel, 2019). Interestingly, voluntary wheel running, also applied in our study, has previously been shown to improve bone status in rodents (e.g., bone thickness, architecture and mineral content) both in physiological (Schlecht et al., 2018) and in different pathological conditions, for example, diabetes mellitus (Minematsu et al., 2017) and ovariectomy‐induced osteoporosis (Fonseca et al., 2011).

There is a growing body of evidence that bone morphogenetic proteins (BMPs)—extracellular cytokines belonging to the TGF‐β superfamily— play an important role in bone remodeling during embryogenesis and in adult life (Deckers et al., 2002). BMPs are specifically involved in the stimulation of osteoprogenitors differentiation into preosteoblastic cells, stimulation of angiogenesis in bones through the production of vascular endothelial growth factor A (VEGF‐A) (David, Feige, & Bailly, 2009; Deckers et al., 2002) and in an increase in osteocalcin and osteopontin expression (Shahi, Peymani, & Sahmani, 2017). Beyond bone repair, BMP superfamily members are involved in cellular and developmental processes including the regulation of the muscle mass (Winbanks et al., 2013), bone‐muscle crosstalk (Ruschke, Hiepen, Becker, & Knaus, 2012), neurogenesis (Mehler, Mabie, Zhang, & Kessler, 1997), and inflammation (Helbing et al., 2017). It has also been demonstrated that among BMP family, BMP4 through upregulation of the calcineurin/nuclear‐factor of activated T‐cells (calcineurin/NFAT) pathway (Shahid et al., 2016) promotes cardiac fibrosis and is involved in pathological cardiac hypertrophy (Sun et al., 2013), which is a leading cause of heart failure. The blockade of BMP4 has thus recently become a potential therapeutic target in this disease (Guo & Dong, 2014).

It is worth emphasizing that a regular physical activity of low‐to‐moderate‐intensity exerts beneficial effects in patients with cardiovascular disease (Dangardt, McKenna, Lüscher, & Deanfield, 2013). However, surprisingly enough, little is known about the impact of physical activity on the expression level of BMPs in heart and bones in heart failure. Therefore, in this study we aimed to evaluate the effect of 8 weeks of spontaneous wheel running activity (mimicking moderate‐intensity exercise) on BMP4 expression in the heart and in the long bones (i.e., tibiae), as well as on the bone phosphorus and calcium content in murine model of heart failure (Tgαq*44 mice). The Tgαq*44 model of transgenic mice results from a cardiac‐specific overexpression of a constitutively active Gαq* protein (Mende et al., 2001). In this model of heart failure, the activation of hypertrophic genes and myocardial fibrosis are evident starting from ~4 months of age, cardiac contractile, and mitochondrial functions begin to deteriorate at 8–10 months of age and finally a clinically evident cardiac decompensation usually occurs at ~12–14 months of age, leading to the animals’ death (Czarnowska et al., 2016; Elas et al., 2008; Mackiewicz et al., 2012; Mende et al., 2001). As presented most recently in our papers (Bardi et al., 2019; Grassi et al., 2017), 8‐week voluntary running exercise is potent to delay chronic heart failure progression in Tgαq*44 mice by decreasing hypertrophy index and by improving cardiac function as reflected by an increase in stroke volume and ejection fraction. Considering the role of BMP4 in the pathological cardiac hypertrophy (Sun et al., 2013) our results might suggest that an improvement of cardiac function in chronic heart failure conditions (Bardi et al., 2019; Grassi et al., 2017) might be BMP4‐dependent. Since an increase in BMP4 expression leads to cardiac hypertrophy and fibrosis through oxidative stress and apoptosis (Sun et al., 2013), a decrease in BMP4 expression in the heart in Tgαq*44 mice after physical training might delay heart failure progression by attenuation of these processes. Interestingly, it has been found that, for example, administration of α‐calcitonin gene‐related peptide delays heart failure progression through the prevention of oxidative stress and apoptosis in the heart of the heart failure mice (Bardi et al., 2019; Kumar, Supowit, Potts, & DiPette, 2019).

## 2 METHODS

Data supporting the findings of this study are available at Jagiellonian University Repository (https://ruj.uj.edu.pl/xmlui/handle/item/152079, https://doi.org/10.26106/nej2-7j88).

### 2.1 Ethical approval

All the experimental protocols were conducted according to the Guidelines for Animal Care and Treatment of the European Union (EU Directive 2010/63/EU for animal experiments), and were approved by the Local Ethics Committee in Krakow (approval No. 37/2013). Please note that all possible steps were taken to minimize animal's pain and suffering. Our studies comply fully with the ethical principles and with journal animal ethics checklist.

### 2.2 Animals

Adult (~10 months old at the start of the study) female FVB wild‐type (WT) and homozygous Tgαq*44 mice were used in the experiment. Both WT and Tgαq*44 mice were subdivided into sedentary (no access to running wheels) and training groups (with access to running wheels). The number of animals were as follows: 13 WT sedentary (WT‐Sed), 14 Tgαq*44 sedentary (Tgαq*44‐Sed), 13 WT trained (WT‐Tre), and 14 Tgαq*44 trained (Tgαq*44‐Tre), respectively.

The model of transgenic mice (Tgαq*44) with chronic heart failure used in our study results from a cardiac‐specific overexpression of a constitutively active Gαq* protein (Mende et al., 2001). 10‐month old Tgαq*44 mice used in the study represent the early phase of heart failure decompensation. The animals utilized in this study were bred at the Institute of Experimental and Clinical Medicine of the Polish Academy of Sciences in Warsaw (Poland). Prior to the experiments the animals were transferred to the animal house at the Faculty of Pharmacy, Medical College, Jagiellonian University in Krakow (Poland). Mice were housed one per cage (floor area of 355 × 235 × 190 mm) and maintained at 22°C–24°C under a 12‐hr light cycle with ad libitum access to water and rodent chow. The training groups were placed in cages equipped with a running wheel allowing to perform voluntary running activity (see below).

### 2.3 Experimental approaches

#### 2.3.1 Running wheel activity

Voluntary wheel running activity of each mouse was recorded continuously using the Running Wheel System (Columbus Instruments Inc.). The system was programmed to record all the running episodes lasting more than 10 s. Mice were also monitored by a digital camera placed in the animal house, allowing the supervising person to check the mice behavior at a given time without disturbing their regular activity. Based on the number of revolutions of the wheel and its radius, the covered distance and the running velocity of the animals were calculated. Data were stored in a computer and downloaded on a weekly basis. The individual data of the covered distance and velocity of running were expressed as a mean ± *SD* value per 24 hr, and were further averaged for the entire period of training (8 weeks, i.e., 56 days).

##### Tissue extraction

Eight weeks after starting the training all the mice (WT, Tgαq*44 from trained and sedentary groups) were sacrificed by a cervical dislocation. Hearts (ventricles) and bones (tibiae) were dissected. Hearts were immediately frozen in the liquid nitrogen (LN_2_). Tibiae were placed in a phosphate‐buffered saline (PBS) and bone marrow cavities were flushed several times with PBS. Next, tibiae were frozen in the LN_2_.

##### Western blot analysis in heart and tibia

**FIGURE 1:**
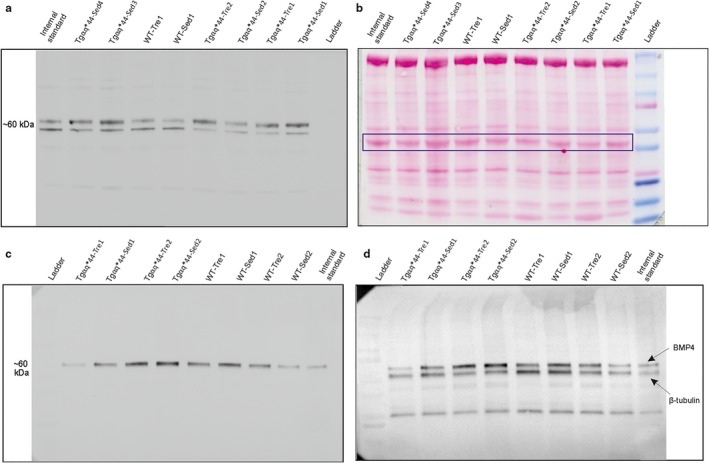
BMP4 detection in the heart and tibia in the wild‐type (WT) mice and in the mice with chronic heart failure (Tgαq*44). Representative immunoblot demonstrating detection of BMP4 protein expression with anti‐BMP4 antibody (Abcam, Cat#ab39973, RRID: AB_2063523) in protein extracts derived from the heart of the WT‐Sed, WT‐Tre, Tgαq*44‐Sed, and Tgαq*44‐Tre mice (panel a) and Ponceau‐S staining of the same membrane demonstrating total protein loaded and bands outlined in violet for normalization of the signal shown in panel a (panel b). Representative immunoblot demonstrating detection of BMP4 protein expression with anti‐BMP4 antibody in protein extracts derived from the tibia of the WT‐Sed, WT‐Tre, Tgαq*44‐Sed, and Tgαq*44‐Tre mice (panel c) and detection of loading control (beta Tubulin, Cat#abE7, RRID: AB_2315513) on the same membrane performed after BMP4 detection (panel d). Protein ladder is a visible Precision Plus Protein Dual Color Standards (Biorad, Cat#1610374). Internal standard in the panel (a) is mouse heart muscle sample and in the panel (c) is mouse tibia sample. WT‐Sed, wild‐type sedentary mice; WT‐Tre, wild‐type trained mice; Tgαq*44‐Sed, Tgαq*44 sedentary mice; Tgαq*44‐Tre, Tgαq*44 trained mice

Heart‐derived cell lysates were prepared using the extraction buffer (62.5 mM Tris pH 6.8, 10% glycerol, 5% SDS), containing protease and phosphatase inhibitor cocktail (Thermo Fisher Scientific™, Waltham, MA, USA, Cat#78415). Tibia was first ground to a fine powder in the LN_2_‐cooled mortars. Subsequently, bone tissue lysate was prepared using the RIPA buffer (Thermo Fisher Scientific™, Waltham, MA, USA, Cat#89900), containing protease and phosphatase inhibitor cocktail (Thermo Fisher Scientific™, Waltham, MA, USA, Cat#78415). Samples were centrifuged for 30 min at 25,000× *g*, the supernatants were transferred into fresh microcentrifuge tubes and stored at −80°C until ready to use. The protein concentration in the sample extracts was measured using a NanoDrop 2000 UV‐Vis Spectrophotometer (Thermo Fisher Scientific™, Waltham, MA, USA, RRID:SCR_015804). The extracts were stored at −80°C until further analysis. Heart‐derived protein extracts (25 µg of total protein) and bone protein extracts (25 µg of total protein) were separated using 4%–20% gradient gels (BioRad, Hercules, CA, USA, Cat#4561093) and then transferred onto nitrocellulose membrane (GE™ Healthcare, Pittsburgh, PA, USA). After transfer Ponceau‐S staining of membranes was performed (Merck KGaA, Darmstadt, Germany, Cat#P7170‐1L). Membranes were subsequently incubated overnight at 4°C with the primary antibodies specific to BMP4 (Abcam, Cambridge, UK, Cat#ab39973, RRID:AB_2063523). To eliminate differences between the gels resulting from the unequal transfer, the internal standard, that is, mouse heart muscle (Figure 1a) and mouse tibia (Figure 1c) was applied on each gel. After the incubation with primary antibody, membranes were washed and incubated in the secondary antibodies conjugated with horseradish peroxidase (Enzo, Life Sciences, Farmingdale, NY, USA, Cat#ADI‐SAB‐300). Chemiluminescent immunoreactive bands were detected by a horseradish peroxidase conjugated secondary antibody (exposure time ~2 min). Data were imaged using GeneGnome 5 Syngene and GeneTools Syngene analysis software was used for densitometric analysis (GenSys 1.2.7.0, Syngene Bio Imaging, Cambridge, UK, RRID:SCR_015770) (Figure 1). Since we have encountered some problems with clear detection of housekeeping proteins such as beta Tubulin (Developmental Studies Hybrydoma Bank, University of Iowa, IA, USA, Cat#abE7, RRID:AB_2315513), GAPDH (Abcam, Cambridge, UK, Cat#ab8245, RRID:AB_2107448) in heart protein lysates and we observed great differences in the signal intensity between wild‐type and Tgαq*44 mice for beta Actin (Abcam, Cambridge, UK, Cat#ab8224, RRID:AB_449644) (Figure 1, Figure S1), we have used Ponceau‐S staining for normalization of protein loading in heart tissue lysates (Figure 1b). Therefore, the optical density values obtained for BMP4 in the heart and tibia were normalized to the internal standard and then either to protein band stained with Ponceau‐S in case of heart muscle (Figure 1b) or beta Tubulin as the loading control in case of bone (Developmental Studies Hybrydoma Bank, University of Iowa, IA, USA, Cat#abE7, RRID:AB_2315513) (Figure 1d). Data were presented in arbitrary unit (a.u.).

**FIGURE 2:**
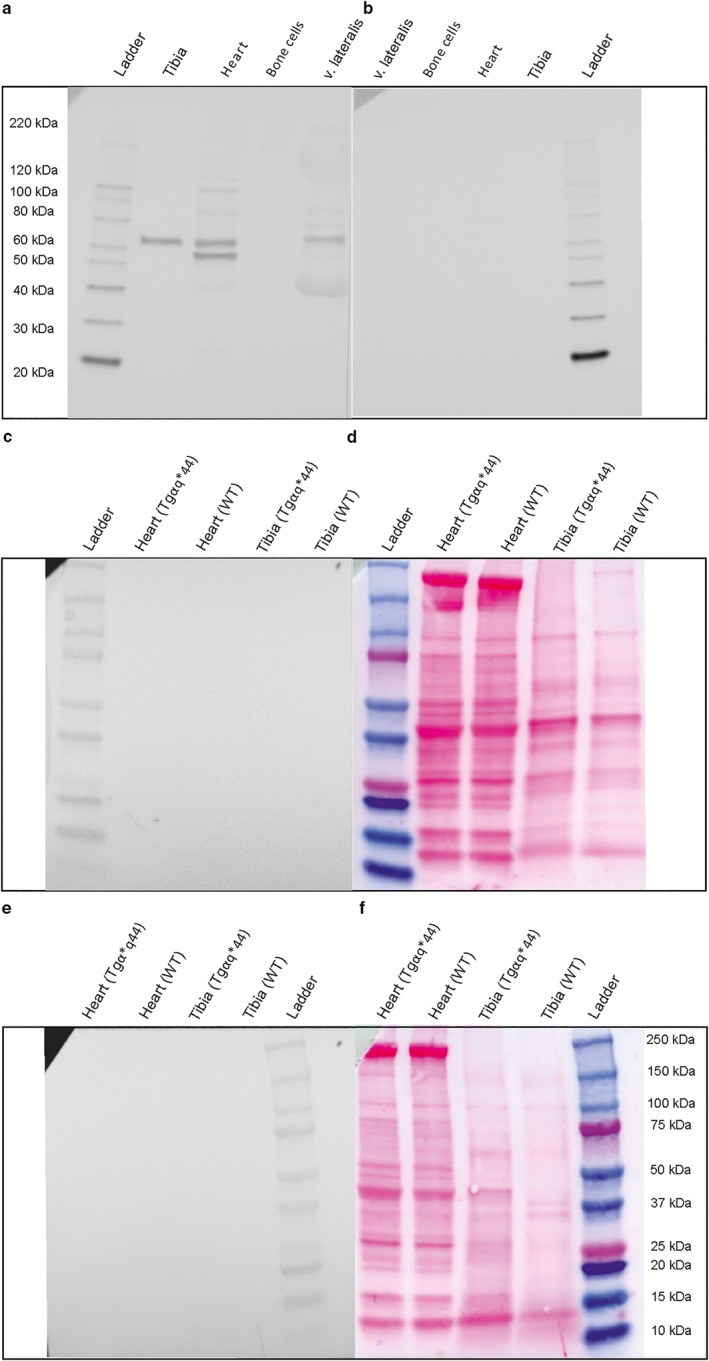
Validation of the anti‐BMP4 antibodies used in the study. Panel (a) Representative immunoblot demonstrating detection of BMP4 expression with anti‐BMP4 antibody (Abcam, Cat#ab39973) in protein extracts derived from: tibia, heart, bone cells and skeletal muscle (vastus lateralis muscle). Panel (b) Representative immunoblot demonstrating loss of the BMP4 expression detection with the antibody ab39973, after blocking the tibia, heart, bone cells, and vastus lateralis muscle‐derived protein extracts with the BMP4 peptide (Abcam, Cat#ab40140). Panel (c) Membrane loaded with protein extracts derived from heart and tibia of sedentary wild‐type (WT) mice and of sedentary mice with chronic heart failure (Tgαq*44) incubated only with anti‐BMP4 antibody and (panel d) Ponceau‐S staining of the same membrane. Panel (e) Membrane loaded with protein extracts derived from heart and tibia of sedentary wild‐type (WT) mice and of sedentary mice with chronic heart failure (Tgαq*44) incubated only with the secondary antibody conjugated with horseradish peroxidase (negative control) and (panel f) Ponceau S staining of the same membrane. Protein standard ladder presented in the panels (a and b) is Magic Mark XP Western Protein Standard (Invitrogen, Cat#LC5602). Protein standard ladder presented in panels (c–f) is a visible Precision Plus Protein Dual Color Standards (Biorad, Cat#1610374)

Specificity of the anti‐BMP4 antibody was confirmed in the another experiment performed with the immunizing human BMP4 peptide (Abcam, Cambridge, UK, Cat#ab40140) which corresponds to the epitope recognized by the anti‐BMP4 antibody (Abcam, Cambridge, UK, Cat#ab39973, RRID:AB_2063523) according to standard Abcam protocol established for target‐specific blockade with immunizing peptide (Figure 2a‐f). We have performed validation of BMP4 antibody using protein extracts derived from tibia, heart, bone cells, and skeletal muscle (vastus lateralis muscle) and comparing the signal obtained for both: human BMP4 immunizing peptide‐blocked and unblocked antibody as a control (Figure 2a‐b).

In our protocol BMP4‐specific bands were visualized at ~60 kDa (Figure 1a,c). However, in the protein extracts derived from hearts both of WT and Tgαq*44 mice additional lower band (~58 kDa) was detected (Figure 1a and Figure 2a). As described by the antibody manufacturer (https://www.abcam.com/bmp4-antibody-ab39973.html) the additional, nonidentified bands can be visualized with this particular antibody. However, it cannot be excluded that the additional lower band (~58 kDa) visible in all heart protein lysates (Figure 1a), but not in bone lysates (Figure 1c) is a potential second heart‐specific BMP4 isoform.

##### X‐ray microanalysis of tibia

The mineral part of bone was determined using electron probe X‐ray microanalysis, which is a widely used method for element determination in biological materials (Warley, 1997). The rest of the elements as well as H, C, N, and O were treated as the remaining mass (RM) of a bone tissue (see below).

For X‐ray microanalysis, tibiae were transferred from the LN_2_ into the brass block cooled down with LN_2_ and slowly warmed up overnight to −80°C, in a vacuum chamber (10^–2^ mBar) of the tissue dryer (ETD4, Edwards High Vacuum International, Burgess Hill, West Sussex, UK). Lyophilization of the samples was performed at the rate of 4°C/hr until the temperature of the specimens reached the room temperature. The bones were then removed from the chamber and gently fractured longitudinally with a razor blade to expose compact part of the bone diaphysis. Next, the samples were attached to the scanning electron microscopy (*SEM*) carbon holder with graphite conductive cement (Ted Pella, Inc., Redding, CA, USA) and coated with 15 nm of carbon (JEOL JEC‐530 auto carbon coater, Tokyo, Japan).

The specimens were analyzed by means of *SEM* combined with Si(Li) energy‐dispersive spectrometer (EDS) (Noran Instruments Inc. Middletown, WI, USA) of 30 mm^2^ crystal size covered with an ultrathin Norvar window. The detector was positioned at take‐off angle of 25°, 30 mm away from the eucentric point of the specimen stage to obtain 0.033 sr solid angle. All the analyses were performed on cortical bones in the raster mode during 100 s of live time and 10 keV accelerating voltage using 470 pA probe current as measured with the Faraday cup. Collected spectra were qualitatively inspected and peak‐to‐background ratios (P/B) of elements (Na, Mg, P, S, Cl, K, and Ca) of interest calculated (Warley, 1997). Quantitative analysis of phosphorus (P) and calcium (Ca) was completed based on the apatite standard (02753‐AB, SPI Supplies, West Chester, PA, USA), measured at the same analytical conditions and calculated using the iteration method as suggested by (Roomans, 1988). The apatite standard was also used to calculate the sensitivity of the EDS measurements on mineralized bone material (ΔC value) (Ziebold, 1967). It has shown that changes in element composition between experimental groups can be considered significant, if the differences in P and Ca are higher than 0.51 and 2.20 mass%, respectively. Three independent raster analyses were performed for each bone specimen to eliminate variations related to roughness of the specimen and/or its chemical heterogeneity. The mean value of those analyses was determined and used for further comparisons between the experimental groups. The number of analyzed tibiae samples was 13 in WT‐Sed, 14 in Tgαq*44‐Sed, 10 in WT‐Tre, and 12 Tgαq*44‐Tre group, respectively.

In this report we decided to apply P/B method for quantitative analysis of elements. First, P/B is the method of choice when organic, biological matrices are investigated. This approach might be further assisted with iterative procedure developed to determine the final concentrations of elements (Roomans, 1988). Second, P/B seem to be the best solution for the analysis of elements in specimens of rough surfaces (Boekestein, Thiel, Stols, Bouw, & Stadhouders, 1984). Thus, P/B was required for the surfaces of bone specimens to be fractured with a razor blade. Since the P/B method followed by the apatite‐based iterative approach was chosen to calculate P and Ca concentrations in bones, it was also possible to determine residual mass (RM) of the bones composed of the organic part (H, C, N, and O) and other mineral and/or biological elements (Na, Mg, S, Cl, and K). Thus, hypothetical protein‐like matrix mixed with inorganic one was considered (H‐6.4%, C‐38.7%, N‐5,8%, and O‐49.1%), and RM fraction calculated iteratively in relation to P and Ca content.

### 2.4 Statistical methods

The results obtained in this study are presented as means, standard deviations (*SD*) and 95% confidence intervals. The data points that deviated from the group means by more than three standard deviations were treated as outliers and excluded from further analysis. In order to analyze the impact of a disease (factor: heart failure, HF) and spontaneous voluntary running (factor: Training) on the BMP4 expression in the heart and tibiae, phosphorus content in tibiae as well as on the body mass, data were analyzed using two‐way ANOVA with post hoc Tukey test performed to identify significant differences between groups. Statistical analyses were performed after checking normality of distribution and homogeneity of variance. In case of BMP4 analysis in heart and in tibiae the original data were transformed to logarithmic scale in order to be able to perform valid analysis of variance. Calcium and residual mass content in tibiae were analyzed using nonparametric Kruskal–Wallis test due to non‐normal data distribution. When comparing the running activity in wheels (total time spent in wheels and total distance during the training period) between WT trained and Tgαq*44 trained mice unpaired, Student’s T‐test was performed. Statistical significance was set at *p* = .05 and the two‐tailed *p*‐values were presented. Statistical analyses were performed using STATISTICA 13.1 (TIBCO Software Inc., RRID:SCR_014213).

## 3 RESULTS

### 3.1 Body mass

No significant differences in the body mass (BM) between subgroups (WT‐Sed, WT‐Tre, Tgαq*44‐Sed, and Tgαq*44‐Tre groups) were observed (*p* > .05). Body mass equaled to 29.5 ± 0.72 g in WT‐Sed, 29.2 ± 0.85 g in Tgαq*44‐Sed, 31.0 ± 0.83 g in WT‐Tre, and 28.3 ± 0.90 g in Tgαq*44‐Tre.

### 3.2 Running activity

**FIGURE 3:**
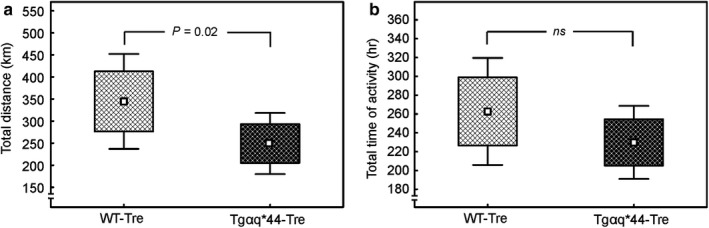
The effect of 8‐week spontaneous running activity on the exercise tolerance in the wild‐type (WT) mice and in the mice with chronic heart failure (Tgαq*44). Total distance (panel a) and total time of activity (panel b) in trained wild‐type (WT‐Tre, *n* = 13) and trained transgenic mouse model of chronic heart failure (Tgαq*44‐Tre, *n* = 14) during 8 weeks of spontaneous wheel running. Boxes and whiskers represent, correspondingly, the 95% confidence intervals for means and the standard deviations. Two‐sided *p*‐values are shown (unpaired Student’s *t* test). WT‐Tre, wild‐type trained mice; Tgαq*44‐Tre, Tgαq*44 trained mice

Total distance covered within 8 weeks of the spontaneous wheel running by the Tgαq*44 mice (249 ± 69 km) was lower by about 28% when compared with the WT mice (345 ± 107 km) (*p* = .02, Figure 3a). Total time spent in the running wheels was lower (by ~ 11%) in the group of Tgαq*44 mice compared to WT mice (229 ± 39 vs. 263 ± 59 hr, respectively, for Tgαq*44 and WT mice), however, this difference has not reached a statistical significance ( *p* > .05, Figure 3b).

### 3.3 BMP4 protein expression in heart and in tibia

**FIGURE 4:**
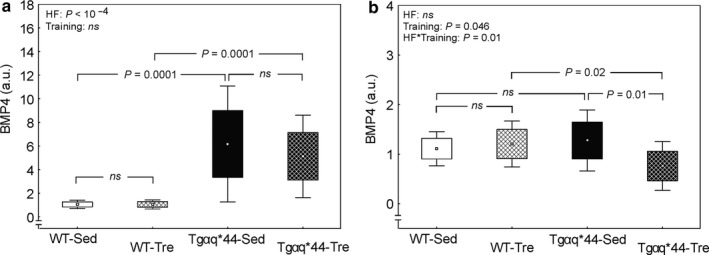
The effect of 8‐week spontaneous running activity on the BMP4 expression in the heart and tibia in the wild‐type (WT) mice and in the mice with chronic heart failure (Tgαq*44). Panel (a) The effect of 8‐week spontaneous wheel running on the BMP4 protein expression in the heart of the WT and Tgαq*44 mice. Boxes and whiskers represent, correspondingly, the 95% confidence intervals for means and the standard deviations. A significant difference between WT‐Sed (*n* = 13) and Tgαq*44‐Sed (*n* = 14) mice as well as between WT‐Tre (*n* = 13) and Tgαq*44‐Tre (*n* = 14) mice is presented (ANOVA, Tukey's post hoc test). Panel (b) The effect of 8‐week spontaneous wheel running on the BMP4 protein expression in the tibia of the WT and Tgαq*44 mice. Boxes and whiskers represent, correspondingly, the 95% confidence intervals for means and the standard deviations. Significant difference between Tgαq*44‐Sed (*n* = 14) and Tgαq*4‐Tre (*n* = 14) mice as well as between WT‐Tre (*n* = 13) and Tgαq*44‐Tre mice (*n* = 14) is presented (ANOVA, Tukey's post‐hoc test). BMP4, bone morphogenetic protein 4; HF, heart failure; T, trained; *ns*, not significant; WT‐Sed, wild‐type sedentary mice; WT‐Tre, wild‐type trained mice; Tgαq*44‐Sed, Tgαq*44 sedentary mice; Tgαq*44‐Tre, Tgαq*44 trained mice

Figure 4 presents analysis of the BMP4 protein expression (the band located at 60 kDa) in the heart (panel A) and tibia (panel B) of wild‐type and chronic heart failure mice. BMP4 protein expression in the heart of the sedentary Tgαq*44 mice (6.17 ± 4.91 a.u.) was significantly higher as compared to the control WT mice (1.05 ± 0.36 a.u.) (by ~490%, *p* = .0001, Figure 4a). Eight weeks of voluntary wheel running had no impact on the BMP4 expression in hearts of either Tgαq*44 or WT mice (*p* > .05, Figure 4a) and after the 8 weeks of voluntary wheel running BMP4 expression in the heart of the Tgαq*44 mice (5.12 ± 3.49 a.u.) was still significantly higher than in the WT mice (1.05 ± 0.39 a.u.) (by ~390%, *p* = .0001, Figure 4a).

Considering the results of the experiment with BMP4 antibody blocked by the immunizing human BMP4 peptide and the lack of both bands (~60 and 58 kDa) in case of heart lysate (Figure 2b) we have additionally analyzed the impact of disease (heart failure; HF) and voluntary wheel running (training) on the sum of upper and lower bands of BMP4 in the heart. We have found, similarly to the results presented in Figure 4a, significant impact of disease (*p* < .0001) and no effect of performed training (*p* > .05) on the BMP4 expression in heart.

BMP4 expression in the tibiae of the sedentary Tgαq*44 mice (1.28 ± 0.62 a.u.) was not significantly different compared to the sedentary WT mice (1.11 ± 0.34 a.u.) (*p* > .05, Figure 4b). Eight‐week voluntary running resulted in a decrease (by ~43%) in BMP4 expression in tibiae of the Tgαq*44 mice (0.76 ± 0.49 a.u.), as judged by a significant interaction between disease and voluntary wheel running (HF*Training, *p* = .01, Figure 4b), whereas BMP4 expression in tibiae of the WT mice remained unchanged after the training. Trained Tgαq*44 mice (0.76 ± 0.49 a.u.), exhibited significantly lower (by ~37%) BMP4 expression in tibiae when compared with the trained WT mice (1.20 ± 0.46 a.u.), (*p* = .02, Figure 4b).

### 3.4 X‐ray microanalysis of tibia

**FIGURE 5:**
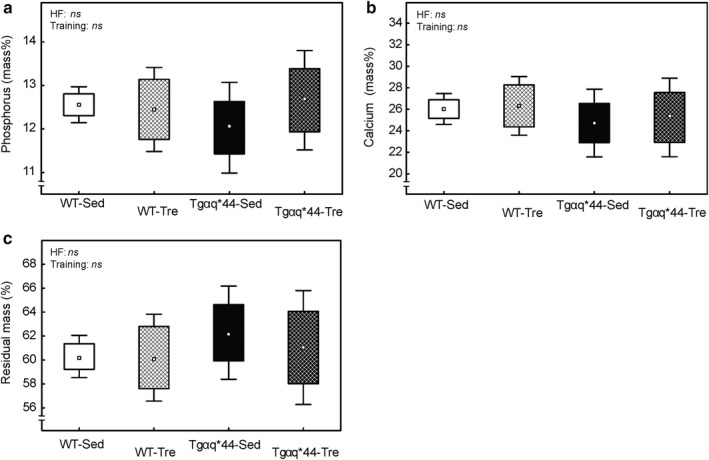
The effect of 8‐week spontaneous running activity on the bone mineral composition and residual mass in the wild‐type (WT) mice and in the mice with chronic heart failure (Tgαq*44). Panel (a) Phosphorus content of tibia of the WT and Tgαq*44 mice. Panel (b) Calcium content of tibia of the WT and Tgαq*44 mice. Panel (c) Residual mass of tibia of the WT and Tgαq*44 mice. Boxes and whiskers represent, correspondingly, the 95% confidence intervals for means and the standard deviations. The number of bone samples per group: WT‐Sed (*n* = 13), WT‐Tre (*n* = 10), Tgαq*44‐Sed (*n* = 14) and Tgαq*44‐Tre (*n* = 12). HF, heart failure; *ns*, not significant; WT‐Sed, wild‐type sedentary mice; WT‐Tre, wild‐type trained mice; Tgαq*44‐Sed, Tgαq*44 sedentary mice; Tgαq*44‐Tre, Tgαq*44 trained mice

X‐ray microanalysis revealed no significant difference in either phosphorus (P), calcium (Ca), or residual mass (RM) in tibiae of the sedentary Tgαq*44 mice compared to the sedentary WT mice (*p* > .05, Figure 5a‐c). No effect of 8 weeks of spontaneous physical activity on the calcium content and residual mass in tibia has either been found (*p* > .05, Figure 5b,c). A higher (by ~6%) phosphorus content present in tibia of the Tgαq*44 trained mice (12.66 ± 1.14 mass%) when compared with their sedentary counterparts (12.03 ± 1.04 mass%) (Figure 5a) has not reached statistical significance (*p* = .17).

## 4 DISCUSSION

In this study we have found that: (a) the BMP4 expression in the heart of the transgenic mouse model of chronic heart failure (Tgαq*4 mice) was about 490% higher than in the heart of the healthy (WT) mice, (b) the spontaneous physical activity of 8 weeks did not affect the BMP4 expression in the heart muscle of the Tgαq*4 mice nor in the WT mice, (c) the performed physical activity decreased the BMP4 content in bone (tibiae) (~43%), but only in the group of Tgαq*44 mice, with no significant effect on the mineral part of bones.

### 4.1 The impact of disease (HF) and long‐lasting physical activity on the BMP4 expression in heart

In this study, we have found significantly higher BMP4 expression in myocardium of Tgαq*44 mice when compared with the wild‐type mice (Figure 4a). With this regard, our results are in agreement with the previous findings showing higher BMP4 expression in pathological cardiac hypertrophy (e.g., Sun et al., 2013). As suggested by these authors, augmentation of BMP4 expression in cardiomyocytes as an effect of pressure overload and/or angiotensin II infusion causes fibrosis and apoptosis mediated via reactive oxygen species production by the NADPH oxidases (Sun et al., 2013) and in consequence leads to cardiac hypertrophy and heart failure. In humans, higher expression of BMP4 was also found in the hearts of patients with coronary artery disease and dilated cardiomyopathy (Pachori et al., 2010; Wu et al., 2014).

The importance of BMP4 signaling in the development of cardiac hypertrophy was demonstrated in studies showing that pressure overload‐induced left ventricular hypertrophy (Sun et al., 2013) and cardiac ischemia–reperfusion injury (Pachori et al., 2010) were attenuated in BMP4‐deficient animals or when BMP inhibitors were used. Increased content of BMP4 in the hearts of Tgαq*44 compared to WT mice both in control and in trained animals (Figure 4a), observed in this study, might be a consequence of the chronic renin–angiotensin–aldosterone (RAA) system activation, which is one of the most important mechanism of the heart failure progression (Hartupee & Mann, 2017). The systemic and tissue RAA system dysregulation has been found to contribute largely to cardiac remodeling, fibrosis, and endothelial dysfunction (Hartupee & Mann, 2017). Recent studies performed on the same animal model of the heart failure (Tgαq*44 mice) demonstrated that the heart failure progression is indeed related to the activation of RAA system and activation of angiotensin converting enzyme/angiotensin II pathway (ACE/Ang II) (Tyrankiewicz et al., 2018). Hence, an increase in the BMP4 content in heart found in this study, which coincides with activation of the renin–angiotensin pathway (Tyrankiewicz et al., 2018), might be an important factor leading to cardiac remodeling during the disease progression. It has also been demonstrated in our previous papers (Bardi et al., 2019; Grassi et al., 2017), that Tgαq*44 mice, at age between 10 and 12 month (at same age as mice in this study) revealed significantly higher heart hypertrophy index, accompanied by an impaired cardiac function. The latter was characterized with significantly lower stroke volume and ejection fraction when compared with healthy animals (WT mice), at the same age. Elevated BMP4 expression in ventricles of the Tgαq*44 mice compared to the control WT mice, observed in our study (Figure 4a), likely results from the RAA system activation. Thus, it may explain higher heart hypertrophy index and impaired cardiac function in Tgαq*44 mice, as presented in our previous papers (Bardi et al., 2019; Grassi et al., 2017).

During our study mice from both experimental groups (Tgαq*44 mice and WT mice) performed relatively large amount of spontaneous physical activity. Namely, the total distance covered by mice reached 249 km (Tgαq*44 mice) versus 345 km (WT mice) and on average they spent about 229 versus 263 hr on running in wheels (respectively for Tgαq*44 mice and WT mice, Figure 3a,b). It should be noticed that the magnitude of the recorded spontaneous physical activity throughout 8 weeks in the mice suffering from the chronic heart failure (Tgαq*44) was significantly lower when compared with control WT mice. Even though the Tgαq*44 mice which were overall physically less active than the WT mice, their daily running distance was 4.5 km on average and they spent more than 4 hr per day on running.

We have found that the magnitude of spontaneous physical activity in wheels in our study had no effect on the BMP4 expression in the heart ventricles in either Tgαq*44 or control WT mice (Figure 4a). Our results indicating no significant effect of physical activity on the BMP4 expression in hearts of the wild‐type mice are in agreement with data reported earlier (Sun et al., 2013). These authors pointed out that even an intense swimming training (4 weeks of training, 90‐min exercise bouts, twice per day, 5 days per week) has no effect on BMP4 expression in heart of the wild‐type mice. Thus, they concluded that BMP4 expression increases only in the pathological (pressure‐overload or angiotensin infusion), but not physiological (physical training) cardiac hypertrophy (Sun et al., 2013).

Interestingly, as presented in our previous study (Grassi et al., 2017), 8 weeks of spontaneous wheel running improved cardiac function of Tgαq*44 mice (an increase in stroke volume and a tendency toward higher ejection fraction), but was inefficient in healthy WT mice. Hence, in view of the no effect of running activity on the BMP4 expression in cardiac ventricles (Figure 4a), an enhancement of cardiac function after 8 weeks of spontaneous wheel running (Grassi et al., 2017) suggests that other factors than BMP4 are involved in improvement of cardiac function after physical activity in the heart failure model of mice (see, e.g., in Mancini et al., 2015). At the same time a significantly higher BMP4 expression in heart of the Tgαq*44 mice (not changed after the spontaneous wheel running) accompanied by an attenuated cardiac function (when compared with WT mice) (Grassi et al., 2017), might explain lower exercise tolerance of the Tgαq*44 mice compared to healthy WT animals in our study (Figure 3). Assuming that, BMP4 indeed plays a pivotal role in mediating the pathological cardiac hypertrophy (Sun et al., 2013), no significant changes in BMP4 expression in the hearts of the Tgαq*44 mice and the WT mice after the 8 weeks of spontaneous physical activity found in our study, clearly suggests that this kind of physical exercise does not increase the risk of development of BMP4‐mediated cardiac hypertrophy.

### 4.2 The impact of disease (HF) and long‐lasting physical activity on the BMP4 expression in bone

It is well‐known that chronic heart failure patients often demonstrate a bone loss and lower bone mineral density as compared to healthy controls (Anker, Clark, Teixeira, Hellewell, & Coats, 1999). In this study, we have not found any significant basal differences in the bone mineral content between WT sedentary healthy mice and Tgαq*44 sedentary mice being a model of chronic heart failure (Figure 5a,b). 8 weeks of spontaneous exercise resulted in a significant decrease in BMP4 protein expression in the tibiae of the Tgαq*44 mice only, without any apparent impact on BMP4 expression in trained WT mice (Figure 4b). Considering the critical role of BMP4 in osteogenesis and angiogenesis (David et al., 2009; Deckers et al., 2002), it might be speculated that the observed impairment in BMP4 expression pattern in tibiae of the trained Tgαq*44 mice might have a negative impact on bone parameters, for example, bone mineral part. However, it is worth emphasizing that the training‐induced attenuation of BMP4 content in tibiae of the Tgαq*44 mice present after 8 weeks of spontaneous wheel running (Figure 4b) had no harmful effect on either the phosphorus, calcium, or residual mass content in tibiae (Figure 5a‐c). Contrary, we have found nonsignificantly higher (by ~6%), phosphorus content after the running activity in cardiac failure (Tgαq*44) mice exposed to running activity (Figure 5a). Therefore, our results show that spontaneous running exercise leading to a decrease in BMP4 in bone (Figure 4b) has no harmful effect on the mineral composition of the bone. On the other hand, this exercise work load appeared not to be sufficient to significantly improve bone mineral density. This is in accordance with earlier reports showing that a low‐to‐moderate intensity, continuous endurance training (similar to the one applied in our study) has minimal effects on bone minerals (Boudenot et al., 2015), whereas a moderate‐intensity interval training or/and training of the heavy intensity, which exerts high load on the musculoskeletal system is more effective in increasing bone mineral density (Boudenot et al., 2015; Trabelsi et al., 2016).

It might be expected that changes in body mass in the time‐course of the physical training could play a role in the magnitude of the mechanical stress exerted on the bone and skeletal muscle tissue both during exercise as well as at rest. However, in this study we have found no significant effect of the performed physical activity on the body mass (Results). Thus, we can exclude the potential effect of the exercise‐induced body mass changes on the observed decrease in BMP4 expression and the mineral status of the bone after the training.

As mentioned earlier, in our previous study we have shown that 8 weeks of a spontaneous wheel running in Tgαq*44 led to an increase in the left ventricular performance (Grassi et al., 2017). In the context of our current experimental setup this effect cannot be explained solely by changes in BMP4 expression in the heart (Figure 4a), however, we cannot neglect a particular role of bone‐derived BMP4 in the cardiac failure conditions and a potential contribution of other tissues, for example, bones to its systemic production. It needs to be mentioned that BMP4 acts as inflammatory cytokine in systemic arteries promoting endothelial activation, endothelial dysfunction, and atherogenesis (Chang et al., 2007; Csiszar, Labinskyy, Jo, Ballabh, & Ungvari, 2008; Helbing et al., 2017; Miriyala et al., 2006). Therefore, a decrease in the bone‐derived BMP4 expression might be beneficial for cardiovascular system. If bones also participate in systemic BMP4 production and thus under a cardiac failure contribute to its negative effects on the vessels, and heart, a decrease in BMP4 levels in tibiae observed in our study after 8 weeks of a moderate physical activity could be a beneficial adaptive mechanism to attenuate cardiac hypertrophy and endothelial dysfunction. However, a systemic role of BMP4 decrease in tibiae, resulting from the physical activity we applied under the cardiac failure conditions still needs to be verified in our ongoing experiments. In addition, the results of this study suggest that the effects of regular physical activity might be varied in physiological versus pathophysiological conditions (WT and Tgαq*44 mice).

It needs to be added that a decrease in BMP4 content after physical activity in tibia in the group of heart failure mice only (Figure 4b), might potentially result from an increase in sclerostin level. As a negative regulator of TGF‐β/BMP and Wnt/β catenin pathways (Asadipooya & Weinstock, 2019), sclerostin is involved not only in bone remodeling, but also (through TGF‐β and Wnt signaling) might play a role in the adverse cardiac remodeling, particularly in the progression of heart failure (Guo & Dong, 2014; Hermans & Blankesteijn, 2015). Higher serum sclerostin concentrations have been found in patients with prevalent cardiovascular disease and were independently associated with cardiovascular mortality (Novo‐Rodríguez et al., 2018). When considering the impact of exercise on the sclerostin level, it has been demonstrated that a single bout of exercise leads to acute increase in blood sclerostin levels (Kouvelioti et al., 2019; Pickering et al., 2017) but several weeks of interval training (Janik, Stuss, Michalska‐Kasiczak, Jegier, & Sewerynek, 2018) decrease blood sclerostin level, which suggests positive, antiosteoporotic effects of a long‐term exercise. However, the impact of regular physical activity on the sclerostin concentration in heart failure condition still remains unknown.

### 4.3 Study limitation

In this paper, we have presented the impact of relatively long‐lasting spontaneous physical activity on the BMP4 content in the heart and bone of the healthy mice and the mice with chronic heart failure. This study was performed using only female mice. It would be worth further investigating, the impact of both the spontaneous and the forced exercise programs using much longer time regimes and compare the effects between females and males of both: WT and Tgαq*44 mice. Moreover, it would be interesting to evaluate the levels of sclerostin as well as Wnt in our bone and heart‐derived extracts. This would certainly shed a new light on our understanding of physiological and molecular cues activated by different exercise regimens, particularly in failing heart.

## 5 CONCLUSIONS

The prolonged period of spontaneous physical activity does not increase the BMP4 content neither in the hearts of the Tgαq*44 nor of the WT mice. However, the applied physical activity lowers down BMP4 content in tibiae in Tgαq*44 with no negative impact on the mineral part of bone. This indicates that spontaneous physical exercise does not increase the risk of the progression of the BMP4–mediated pathological cardiac hypertrophy. On the other hand, given the proinflammatory function of BMP4, training‐induced decrease in the BMP4 content in bone might be beneficial for the failing heart but further studies are needed to better understand the link between bone metabolism and heart failure.

## CONFLICT OF INTEREST

None declared.

## AUTHOR CONTRIBUTIONS

J.M. conception and design of the study, funding acquisition, acquisition of data, analysis and interpretation of data, writing the original draft of the manuscript; J.F. acquisition of data, analysis and interpretation of data, writing the original draft of the manuscript; G.T. acquisition of data, analysis and interpretation of data, revising the manuscript; M.G., J.K., E. Piechowicz., E. Pyza. acquisition of data, revising the manuscript; S.Ch. analysis and interpretation of data, revising the manuscript; J.A.Z. funding acquisition, analysis and interpretation of data, revising the manuscript. All authors approved the final version of manuscript and agree to be accountable for all aspects of the work in ensuring that questions related to the accuracy or integrity of any part of the work are appropriately investigated and resolved. All persons designated as authors qualify for authorship, and all those who qualify for authorship are listed.

## Supporting information



Fig S1
