# Supplementary figures and images for: Impact of long‐lasting spontaneous physical activity on bone morphogenetic protein 4 in the heart and tibia in murine model of heart failure
Source: Physiol Rep. 2020 Apr 21;8(8):e14412. doi: 10.14814/phy2.14412 (PMC7174143; doi:10.14814/phy2.14412)

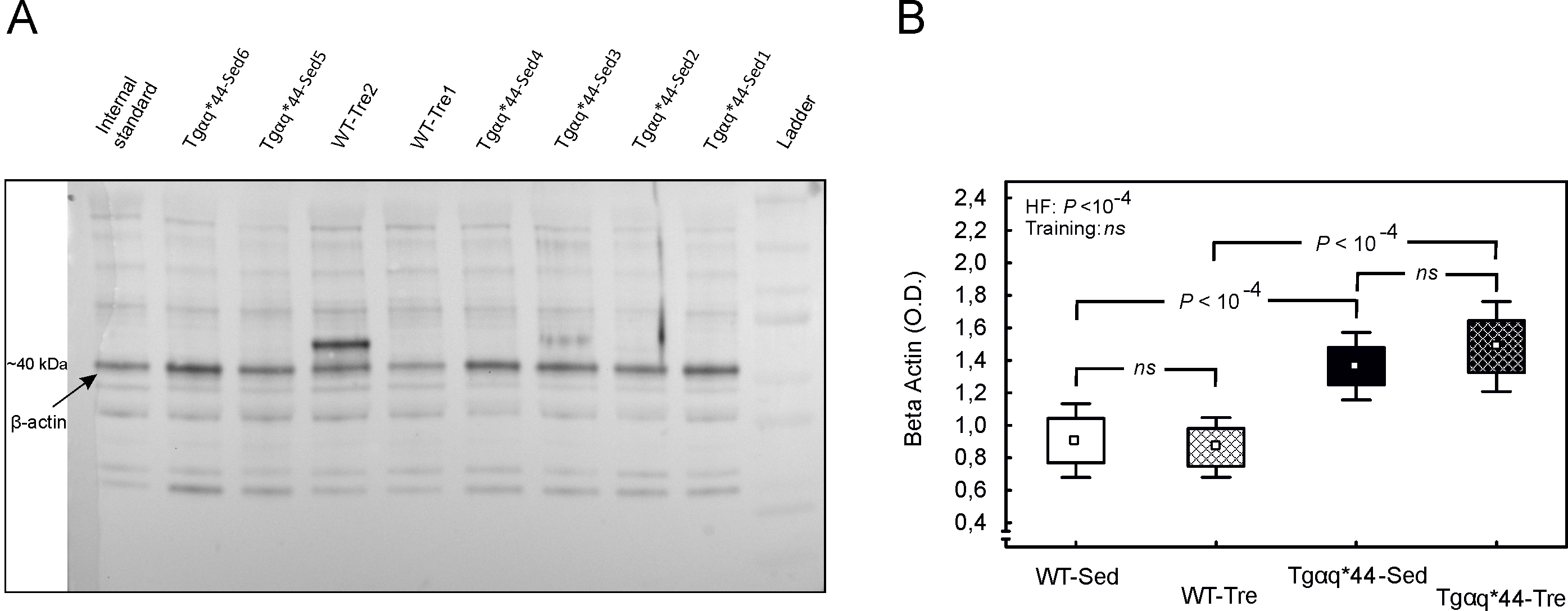

Supplement: Supplementary file 1 — Fig S1 [file PHY2-8-e14412-s001.tif]
